# Primary Undifferentiated Pleomorphic Thyroid Sarcoma Presenting as Superior Vena Cava Syndrome: A Case Report

**DOI:** 10.7759/cureus.20104

**Published:** 2021-12-02

**Authors:** Soroush Shahrokh, Mohadese Shahin, Ramin Malboosbaf, Nasrin Shayanfar

**Affiliations:** 1 Graduate Medical Education, HCA Houston Healthcare Kingwood/University of Houston School of Medicine, Kingwood, USA; 2 Radiation Oncology, Iran University of Medical Sciences, Tehran, IRN; 3 Endocrinology and Metabolism, Iran University of Medical Sciences, Tehran, IRN; 4 Pathology, Iran University of Medical Sciences, Tehran, IRN

**Keywords:** undifferentiated thyroid sarcoma, undifferentiated pleomorphic thyroid sarcoma, superior vena cava syndrome, thyroid sarcoma, primary thyroid sarcoma, metastatic thyroid carcinoma, metastatic thyroid cancer, thyroid carcinoma, thyroid cancer

## Abstract

Primary thyroid sarcomas (PTS) are an incredibly uncommon type of thyroid cancer. Undifferentiated pleomorphic sarcomas of the thyroid (UPS-T) are extremely rare subtypes of thyroid sarcoma with no defined cell differentiation. Here, we report the case of a 60-year-old female with a two-year history of hypothyroidism who presented to our hospital with productive cough, dyspnea, and diffuse facial edema for two weeks. Her chest computed tomography (CT) scan revealed a large anterior mediastinal mass and multiple bilateral pulmonary nodules. Her thyroid ultrasound showed two hypoechoic nodules, while a CT-venogram of the right upper extremity showed superior vena cava and the right brachiocephalic vein obstruction, which was relieved with angioplasty. A biopsy of the anterior mediastinal mass showed poorly differentiated pleomorphic thyroid sarcoma. The patient was not a candidate for inpatient chemo- or radiotherapy because of her overall medical condition. One week later, she developed worsening respiratory failure, was intubated and transferred to the intensive care unit (ICU), where she passed away two days later.

## Introduction

Thyroid carcinomas are among the most common malignancies in the United States (US), with an estimated 1.2% lifetime risk of diagnosis [1-2]. The main subtypes of thyroid carcinomas include papillary, follicular, Hürthle cell, medullary, and anaplastic carcinomas [3]. Papillary thyroid tumors are the most common and account for approximately 90% of all thyroid neoplasms, while anaplastic thyroid carcinomas are the least common with an incidence of approximately 0.8% [2]. Other primary neoplasms of the thyroid include lymphomas and sarcomas, although rare, with primary thyroid sarcomas (PTS) incidence ranging from as low as 0.01% to 1.5% of all thyroid malignancies [4-7].

Here, we describe a rare case of a patient with undifferentiated pleomorphic sarcoma of the thyroid (UPS-T) presenting as superior vena cava syndrome (SVCS). UPS-T are extremely rare, and to the best of our knowledge, this is one of the first reported cases of UPS-T presenting as an SVCS.

## Case presentation

A 60-year-old female with a two-year history of hypothyroidism on 100 mcg levothyroxine daily and osteoarthritis of the cervical spine presented to the hospital with productive cough and dyspnea for two weeks. She also developed diffuse facial edema and orthopnea for the past four days. She denied any associated fever, myalgias, anorexia, nausea, or vomiting. She had no history of first or second-hand exposure to tobacco. On admission, her vital signs were significant for tachypnea with a respiratory rate of 28 breaths-per-minute and hypoxia with an oxygen saturation of 90% on room air. She was placed on 4L of nasal cannula supplemental oxygen, with her oxygen saturation increasing to 98%. Her physical exam was remarkable for diffuse facial plethora, moderately enlarged thyroid gland with nodularity on the left side presenting as a firm, non-tender neck mass, and bibasilar crackles in the bilateral lower lungs.

Her laboratory studies showed leukocytosis, mild transaminitis, elevated lactate dehydrogenase, hypoalbuminemia. Her arterial blood gas (ABG) on 4L of nasal cannula supplemental oxygen was within normal range with normal oxygenation. Her thyroid function tests showed a low thyroid-stimulating hormone (TSH) level but an otherwise normal triiodothyronine (T3) and thyroxine (T4) levels (Table 1).

**Table 1:** Laboratory study results. TSH: thyroid-stimulating hormone; PT: prothrombin time; PTT: partial thromboplastin time; INR: international normalized ratio; ALT: alanine transaminase; AST: aspartate transaminase; ALP: alkaline phosphatase; LDH: lactate dehydrogenase; T3: triiodothyronine; T4: thyroxine; PaCO2: partial pressure of carbon dioxide; PaO2: partial pressure of oxygen; HCO3: bicarbonate; Mg: magnesium; PO: phosphate; Ca: calcium; Bili(T): total bilirubin; Bili(D): direct bilirubin; Hb: hemoglobin; Plt: platelets; WBC: white blood cells

Laboratory Study	Results	Reference Range
WBC	17.8 x 10^3/mm3	4.5-11.0 x 10^3/mm3
Hb	12.3 x 10^3/mm3	12.0-15.5x 10^3/mm3
Plt	425 x 10^3/mm3	150-350 x 10^3/mm3
PT	13 sec	10-12 sec
PTT	32 sec	30-45 sec
INR	1	
ALT	66 U/L	0-35 U/L
AST	70 U/L	0-35 U/liter
ALP	241 U/L	30-120 U/liter
Bili(T)	0.8 mg/dl	0.3-1.0 mg/dl
Bili(D)	0.2 mg/dl	0.1-0.3 mg/dl
Albumin	3.2 g/dl	3.5-5.5 g/dl
Ca	9.7 mg/dl	9.0-10.5 mg/dl
PO	4.1 mg/dl	3-4.5 mg/dl
Mg	2.3 mg/dl	1.8-3.0 mg/dl
LDH	1,336 U/liter	100-190 U/liter
pH	7.45	7.35-7.45
PaCO2	33.4 mm Hg	35-45 mm Hg
HCO3	23.5 mEq/L	21-30 mEq/L
PaO2	79.4 mm Hg	80-100 mm Hg
TSH	0.4 µU/ml	0.5-4.7 µU/ml
Total T3	157 ng/dl	60-181 ng/dl
Total T4	10.8 µg/dl	4.5-10.9 µg/dl

**Figure 1:**
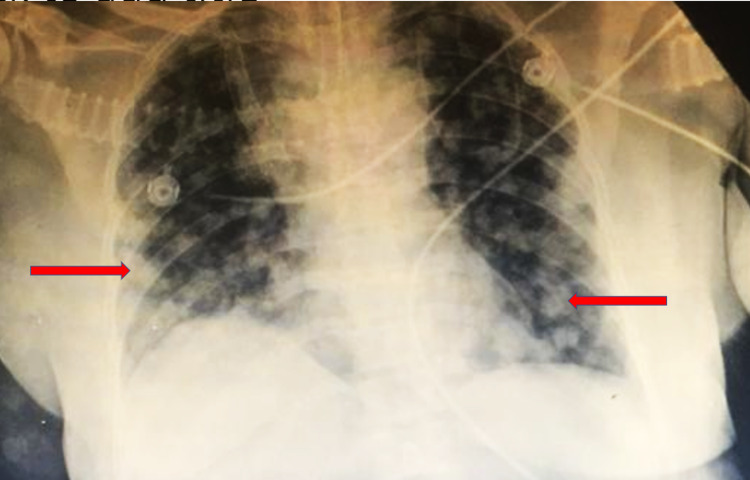
Patient’s chest X-ray on arrival showed multiple nodular opacities in bilateral lungs (as indicated with the red arrows).

**Figure 2:**
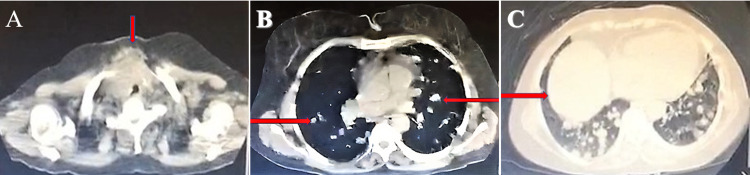
Patient's spiral chest CT scan showed (A) two nodules in the isthmus of the thyroid gland (as shown by the red arrow), (B) multiple cotton wool nodules in the parenchyma of the bilateral lungs (as shown by the red arrows), and (C) a hypodense lobular mass at the thoracic inlet extending into the anterior mediastinum (as shown by the red arrow), consistent with metastatic disease.

**Figure 3:**
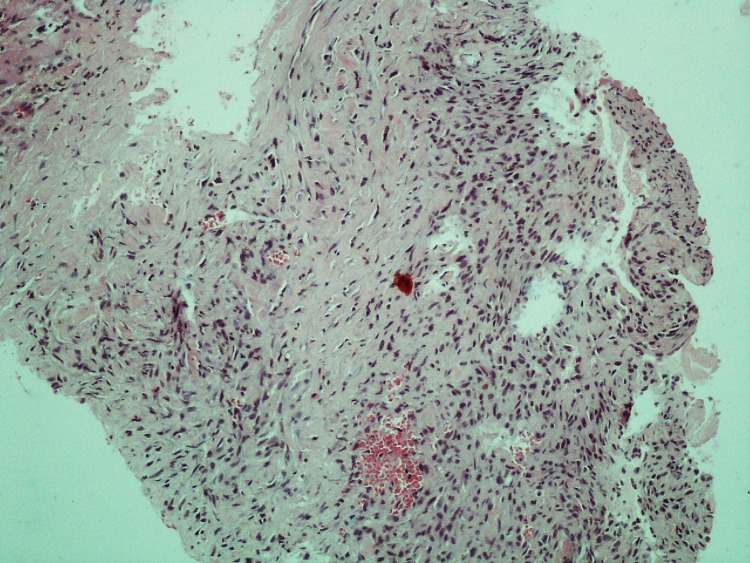
H&E stain of the patient’s anterior mediastinal biopsy (x10 magnification) showed follicles of spindle cells with hyperchromatic irregular nuclei. H&E: Hematoxylin and Eosin

**Figure 4:**
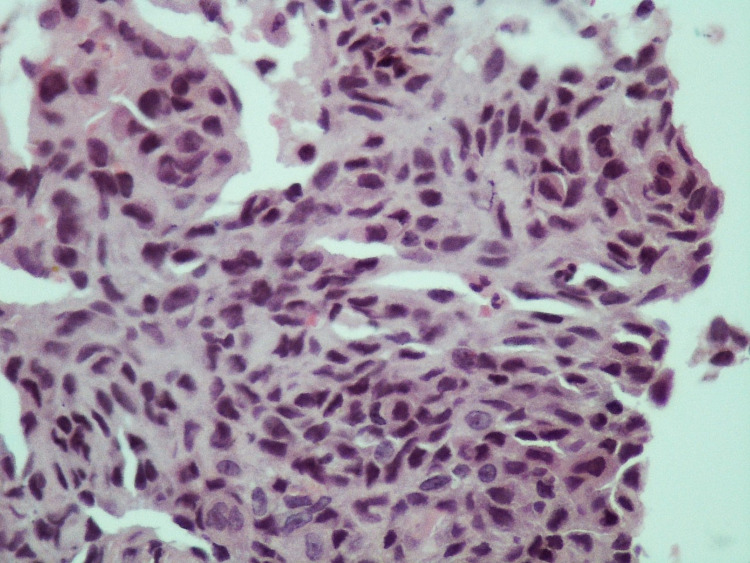
H&E stain of the patient’s anterior mediastinal biopsy (x40 magnification) shows follicles of spindle cells with hyperchromatic irregular nuclei and inconspicuous nucleoli, along with indistinct cytoplasm set in a collagenized stroma, consistent with poorly differentiated neoplastic spindle cells, which was in favor of spindle cell neoplasm of the thyroid compatible with poorly differentiated thyroid sarcoma.

The patient had a doppler ultrasound of the thyroid, which showed the right thyroid lobe measuring 3.9x1.6x1.5-cm and the left lobe measuring 3.8x1.8x1.4-cm, along with two hypoechoic nodules measuring 2.1x2.2-cm 1.8x1.4-cm in the isthmus. The patient’s chest X-ray (CXR) revealed multiple nodular opacities throughout the bilateral lung fields (Figure 1). Computed tomography (CT) scan of the chest without intravenous (IV) contrast confirmed the presence of the thyroid nodules (Figure 2A), and revealed a large anterior mediastinal mass (Figure 2B) and multiple bilateral pulmonary nodules, most likely metastatic disease (Figure 2C). CT-venogram of the right upper extremity showed 70% obstruction of the superior vena cava (SVC) and almost 100% obstruction of the right brachiocephalic vein. The patient underwent angioplasty of the right upper extremity and superior vena cava, which entirely relieved the obstruction. The patient's echocardiogram showed a normal left ventricular (LV) function with an ejection fraction of 55% but mild grade 1 LV diastolic dysfunction. It also showed mild pericardial effusion anterolateral to the right ventricle with a 7-mm fibrin cap but without significant hemodynamic effects. Furthermore, it showed a large heterogenous vascular mass in the anterior side of the aortic arch contributing to its deviation with significant narrowing and resultant turbulent flow of the SVC, suggestive of tumor invasion. The patient underwent a CT-guided biopsy of the anterior mediastinal mass. Microscopic examination and immunohistochemical (IHC) study of the mass showed poorly differentiated neoplastic spindle cells consistent with poorly differentiated thyroid sarcoma (Figures 3, 4).

The patient was evaluated by endocrinology, cardiology and hematology, and oncology services. She was placed on oral furosemide 40-mg twice a day and oral clopidogrel 75-mg daily for antiplatelet therapy. She was determined to be a poor candidate for inpatient chemo- or radiotherapy due to her high oxygen requirements and overall critical condition. The patient's hospital stay was complicated by increasing oxygen requirements and the development of acute kidney injury. Approximately one week after hospitalization, the patient had worsening hypoxia with a significant drop in her oxygen saturation to 90% on continuous bilevel positive airway pressure (BiPAP) machine with 100% fraction of inspired oxygen (FiO2). She was intubated with mechanical ventilation and transferred to the intensive care unit. Her ICU stay was complicated by hypotension, for which she was placed on epinephrine and norepinephrine drips. She also developed progressively worsening respiratory failure and renal failure while in the ICU. Unfortunately, she had a cardiac arrest on the second day of her ICU stay and passed away.

## Discussion

Sarcomas are tumors that arise from the malignant transformation of mesenchymal cells. They are classified inversely according to their level of differentiation, with well-differentiated tumors considered low-grade, moderately differentiated tumors as intermediate-grade, and poorly differentiated or undifferentiated tumors considered high-grade sarcomas [8]. PTS is rare thyroid cancer, with a reported incidence ranging from 0.01% to 1.5% of all thyroid malignancies [4-7]. While the exact etiology of these tumors remains unknown, some studies have reported a history of head and neck radiation in patients diagnosed with PTS [9].

The main subtypes of PTS include angiosarcoma, hemangioendothelioma, leiomyosarcoma, fibrosarcoma, osteosarcoma, liposarcoma, and undifferentiated pleomorphic sarcoma (UPS-T) [6-7]. UPS-T, previously known as malignant fibrous histiocytoma (MFH), is an extremely rare subtype of thyroid sarcoma without any defined cell differentiation [10]. As PTS are extremely rare, they have a high rate of misdiagnosis, especially as anaplastic thyroid carcinomas. While PTS has no specific clinical signs or symptoms, it generally presents as a rapidly enlarging, firm, non-tender neck mass, which may be accompanied by acute onset of cough, dyspnea, orthopnea, and dysphagia due to compression of the trachea or esophagus, respectively [7]. These clinical findings were consistent with those seen in our patient, who presented with acute onset of cough and dyspnea, which progressed to acute hypoxic respiratory failure within less than three weeks.

Thyroid ultrasound and CT scan may be a valuable tool in differentiating PTS and other types of thyroid cancers, including anaplastic thyroid carcinomas [7,11]. In PTS, thyroid ultrasound typically shows multiple hypoechoic nodules with regular borders with an otherwise normal gland, while ultrasound of the anaplastic thyroid carcinomas typically shows nodules with irregular borders. Furthermore, CT scan shows hypodense nodules [7,11]. This was also the case in our patient, who had two hypoechoic nodules in the thyroid isthmus but an otherwise normal thyroid gland. Furthermore, her chest and mediastinal CT scan showed multiple hypodense lesions. The gold standard for diagnosis of PTS tumors is histopathologic examination with immunohistochemical staining. On microscopic exam, PTS is classically composed of plump spindle cells arranged in a storiform pattern with high pleomorphism and giant cells [7,10].

PTS is generally not associated with lymph-node invasion or distant metastasis [7,12,13,14,15]. In a systematic review of PTS, Surov et al. found that 73% of patients had no lymph node or distant organ metastases at the time of diagnosis. In patients with tumor dissemination to extra-thyroid tissues, lymph node involvement was seen in only 6.3% of patients, the majority of whom were diagnosed with primary thyroid fibrosarcoma. Meanwhile, patients with thyroid histiocytoma and liposarcoma typically developed adjacent tumor infiltration of the trachea or the esophagus. Thyroid leiomyosarcoma, osteosarcoma, and angiosarcoma, meanwhile, were commonly associated with distant organ metastasis, most often involving the lungs [7]. This stands in contrast to our patient, who presented with severe signs of distant metastasis, including SVC syndrome and severe dyspnea, and developed respiratory failure because of bilateral lung metastasis, which eventually led to her demise.

While thyroid neoplasms generally have excellent outcomes with a five-year survival rate greater than 98.3% [1-2], PTS has a grim prognosis [7,10]. Because of the rarity of these tumors, there is no consensus on the best treatment approach. However, surgery plays a central role in treating these tumors, with early and radical thyroidectomy typically associated with favorable outcomes, with a local recurrence rate of 27% within five years, compared to 86% seen with marginal resection [10,12-14]. However, negative margin surgical resection is a difficult task given the complexity of head and neck anatomy. In general, lymph node dissection is not recommended for these tumors, as PTS rarely metastasize to the regional lymph nodes [13]. Because of high recurrence rates associated with partial surgical resection alone, neoadjuvant and adjuvant chemoradiation is also often used to treat these tumors, despite a lack of statistically significant improvement in outcomes [10]. Nguyen et al. analyzed 21 cases of primary undifferentiated pleomorphic sarcoma treated with surgery and adjuvant chemoradiation. Their analysis showed no improvement in disease-free survival or decrease in tumor burden associated with adjuvant chemoradiation when compared to surgical resection alone. However, these results may have been due to the small sample size [10]. On the other hand, in a retrospective study, Huber et al. reported improved outcomes and disease-free survival in patients with sarcomas of the head and neck larger than 4-cm who received adjuvant radiotherapy [15].

## Conclusions

Thyroid cancers are a relatively common malignancy in the US. Meanwhile, PTS, and especially UPS-T, are extremely rare. Here, we describe a patient with a history of hypothyroidism diagnosed with UPS-T presenting as multiple lung metastases and a large anterior mediastinal mass leading to SVCS. To our best knowledge, this is the first reported case of UPS-T in a patient with a history of hypothyroidism presenting with SVCS. This report emphasizes the importance of considering PTS in patients with a rapidly enlarging neck mass with the typical ultrasound findings, as PTS, and UPS-T, in particular, may quickly progress to metastatic disease.
